# Patients with Asthma and Comorbid Allergic Rhinitis: Is Optimal Quality of Life Achievable in Real Life?

**DOI:** 10.1371/journal.pone.0031178

**Published:** 2012-02-17

**Authors:** Fulvio Braido, Ilaria Baiardini, Stefania Menoni, Federica Gani, Gian Enrico Senna, Erminia Ridolo, Veruska Schoepf, Anthi Rogkakou, Giorgio Walter Canonica

**Affiliations:** 1 Allergy and Respiratory Disease Clinic, Ospedale S. Martino Genoa, Genoa, Italy; 2 Biostatistic Unit, Department of Health Science, University of Genoa, Genoa, Italy; 3 Allergy Unit, Respiratory Diseases Division, A.O.U. San Luigi, Orbassano, Torino, Italy; 4 Allergy Service, Verona Major Hospital, Verona, Italy; 5 Clinical Sciences, University of Parma, Parma, Italy; Tulane University, United States of America

## Abstract

**Objectives:**

Asthma trials suggest that patients reaching total disease control have an optimal Health Related Quality of Life (HRQoL). Moreover, rhinitis is present in almost 80% of asthmatics and impacts asthma control and patient HRQoL. We explored whether optimal HRQoL was reachable in a real-life setting, and evaluated the disease and patient related patterns associated to optimal HRQoL achievement.

**Methods and Findings:**

Asthma and rhinitis HRQoL, illness perception, mood profiles, rhinitis symptoms and asthma control were assessed by means of validated tools in patients classified according to GINA and ARIA guidelines. Optimal HRQoL, identified by a Rhinasthma Global Summary (GS) score ≤20 (score ranging from 0 to 100, where 100 represents the worst possible HRQoL), was reached by 78/209 (37.32%). With the exception of age, no associations were found between clinical and demographic characteristics and optimal HRQoL achievement. Patients reaching an optimal HRQoL differed in disease perception and mood compared to those not reaching an optimal HRQoL. Asthma control was significantly associated with optimal HRQoL (χ^2^ = 49.599; p<0.001) and well-controlled and totally controlled patients significantly differed in achieving optimal HRQoL (χ^2^ = 7.617; p<0.006).

**Conclusion:**

Approximately one third of the patients in our survey were found to have an optimal HRQoL. While unsatisfactory disease control was the primary reason why the remainder failed to attain optimal HRQoL, it is clear that illness perception and mood also played parts. Therefore, therapeutic plans should be directed not only toward achieving the best possible clinical control of asthma and comorbid rhinitis, but also to incorporating individualized elements according to patient-related characteristics.

## Introduction

The goal of asthma treatment is to reach the good symptom control and to prevent disease worsening [1], leading to an improvement of Patient-Reported Outcomes (PROs). Among these, Health-Related Quality of Life (HRQoL) is a widely used outcome to assess patients' perspective [2].

Clinical trials have demonstrated that total asthma control is an achievable target at all disease stages [3], [4], and that HRQoL correlates with the level of disease control [5]. Furthermore, when treatment is tailored to achieve total control, the vast majority of asthma patients (regardless of the severity of asthma) have the possibility of reaching HRQoL scores approaching the maximum, with little or no impact of the disease on patient's life [6].

Results from the above trials refer to asthma patients studied in the framework of controlled clinic trials and the study design did not take comorbid rhinitis into account. Observational studies indicated that patients suffering from symptomatic rhinitis were less likely to attain good asthma control and emphasised that the presence of rhinitis symptoms influenced not only the quality of life related to the upper airways but also to lower airways and the global HRQoL score [7]–[11]. Up to now, the possibility of patients with asthma and comorbid rhinitis to reach an optimal HRQoL in real life has not been explored. We speculated that this goal is achievable and that it could be associated to a different disease experience. Reaching an optimal HRQoL, from patient's perspective, means to minimize or even to cancel the impact of the disease on daily life. To date this aspect has not been evaluated. We speculated that when optimal HRQoL is achieved, patients' generate new cognitive representations of, and emotional reactions to, their illness. In other words, if the HRQoL is minimum, the disease could be considered [12].

The aims of our cross-sectional study were: to assess the level of quality of life related to asthma and rhinitis in an adult population in order to evaluate whether optimal HRQoL was achievable in a real-life setting; to investigate the clinical and demographic pattern associated to optimal HRQoL achievement; to assess whether patients reaching optimal HRQoL differ in terms of illness perception and stress levels from those who do not; to investigate whether among the patients with controlled asthma, those reaching an optimal HRQoL differ in illness perception and stress levels.

## Materials and Methods

### Patients

Asthma and rhinitis patients scheduled for a follow-up visit to the Allergy and Respiratory Departments of six Italian medical centers were recruited over a two-month period starting in November 2009. The descriptive observational study was approved by the Ethics Committee of the Azienda Ospedaliero-Universitaria San Martino di Genova and written informed consent was obtained from each patient prior to the beginning of the study.

Inclusion criteria for the study were: adult age (18–75 years), comprehension of spoken and written Italian, availability to participate in the study (informed consent signature), and a history of diagnosed allergic asthma with concomitant rhinitis [13], [14]. Exclusion criteria were: presence of impaired cognitive functions, visual-auditory deficits, and clinical conditions incompatible with questionnaire completion.

While the patients were waiting for their follow-up visit, they were invited to complete the required questionnaires (see below). During the visit, the ARIA guidelines were used to establish the patient's rhinitis status [13]. Taking into account the patients were classified according to GINA guidelines [14]. For the current study, the patient's level of control (ACT score [15]) and therapeutic strategy were recorded.

### Questionnaires

The Asthma Control Test (ACT) [15], [16] permits the determination of the level of asthma control. This questionnaire consists of five items that investigate the presence, in the preceding four weeks, of limitations at work or school due to asthma, the presence of diurnal and nocturnal symptoms, and the use of rescue medications. One item concerns the subjective perception of the level of asthma control. Patients assigned scores of 1–5 to each item, resulting in the following grading system: uncontrolled asthma, scores 5–19; well controlled asthma, scores 20–24; total disease control, score of 25.

Rhinitis symptoms were assessed using the Total 5 Symptoms Score (T5SS) that includes the following symptoms: nasal discharge (rhinorrhea), nasal congestion, itchy nose, sneezing, and itching eyes. All symptoms were graded using a score from 0 (absent) to 3 (very troublesome), with total scores ranging from 0–15.

The RHINASTHMA is the only questionnaire that allows the evaluation of the impact of concomitant rhinitis and asthma on patient HRQoL [17]. RHINASTHMA is composed of 30 items; the analysis provides individual scores for upper airways, lower airways, respiratory allergy impact, and a composite score, the Global Summary (GS), which indicates the overall impact of the disease. Patients use a five-point Likert scale (not at all, a little, fairly, much, very much) to indicate the extent to which they were bothered by each problem during the two weeks preceding the completion of the questionnaire. These responses were then converted into scores from 0 to 100, with higher scores corresponding to worse HRQoL. Similar to previous studies in which optimal HRQoL was defined as Asthma Quality of life (AQLQ) score of 80% or higher [6], a RHINASTHMA GS score from 0 to 20, indicating minimal or absent disease impact on patient life, was considered reflective of optimal HRQoL.

The Illness Perception Questionnaire (IPQ-R) is a widely-used questionnaire that assesses patient beliefs and understanding of the illness [18]. The “identify factor” section asks the patient about the presence of symptoms (pain, sore throat, nausea, fatigue, weight loss, stiff joints, sore eyes, breathlessness, headache, upset stomach, sleep disturbances, dizziness, and loss of strength) and whether the patient believes that each selected symptom is related to asthma/rhinitis. The “identity factor” score is calculated as the sum of the scores for these symptoms. A five-point Likert scale (1 = strongly disagree, 5 = strongly agree) is employed in the second section of the IPQ-R, which investigates the following disease-related symptoms: consequences, timeline acute/chronic, timeline cyclical, coherence, personal control, treatment control, and emotional representation. The factors are calculated as the sum of the item scores.

The Profile of Mood States (POMS) is a well-established, factor-derived measure of psychological distress [19]. The test consists of 58 adjectives to which patients respond on a five-point scale (0 = not at all to 4 = extremely), providing a global index of stress and six factorial scores: Tension-Anxiety, Depression-Dejection, Anger-Hostility, Vigor-Activity, Fatigue-Inertia, and Confusion-Bewilderment. The scores for each scale were normalized to a mean of 50 and a standard deviation of 10.

### Statistical analysis

Patients were included in the statistical analysis on the basis of completeness of clinical data and completion of >90% in the PRO questionnaires. Data are presented in percentage for demographic characteristics and clinical data, as mean ± standard deviation for continuous variables investigated. Exploratory analysis of demographic and clinical data was performed using the following procedures. Pairwise associations between categorical variables were assessed through the chi-squared test for heterogeneity. Student's *t*-test was used to detect significant differences in the means of quantitative variables for independent samples. Spearman's rho was used to assess the relationships between variables or rank scores. Discriminant power of the POMS total score on optimal HRQoL was assessed through receiver operating characteristic (ROC) analysis. To estimate the association of each IPQ-R factor with RHINASTHMA GS scores, a linear regression model was applied (statistical significance: α = 0.05).

## Results

Out of 238 patients initially recruited for the study, 227 agreed to fill in the questionnaires. Eighteen patients were excluded from the analysis due to clinical data incompleteness or missing questionnaire answers (>10%), leaving 209 patients (mean age 45.14±16.75 years) in the final analysis group (Table 1). ACT and T5SS mean scores showed far-from-ideal asthma and rhinitis control (Table 2). HRQoL was also impaired, reaching only 70% of the best score.

**Table 1:** Demographic characteristics and clinical data for the 209 patients in the final analysis group.

DEMOGRAPHIC CHARACTERISTICS		
**Sex**	Male	36.5%
	Female	63.5%
**Smoking**	Smoker	16.5%
	Non-smoker	72.9%
	Former smoker	9.6%
**Education**	Primary school	9.3%
	Secondary school	19.5%
	High school	47.8%
	Academic degree	23.4%

CLINICAL DATA			
**Rhinitis**	**Intermittent**Persistent		51.8%48.2%
	MildModerate to severe		51.9%48.1%
**Asthma**	GINA Classification in therapy	12345	22.7%29.5%36.9%9.7%1%

**Table 2:** Asthma and rhinitis related quality of life, rhinitis symptoms and level of control expressed as minimal, maximal and mean scores in observed population (n = 209).

	Minimum	Maximum	Mean	Std. Deviation
**RHINASTHMA UA**	0	100.00	31.6025	23.54
**RHINASTHMA LA**	0	84.62	30.5953	21.77
**RHINASTHMA RAI**	0	87.50	25.7852	20.27
**RHINASTHMA GS**	0	83.33	29.6147	18.56
**T5SS TOT**	0	15	7.01	4.04
**ACT TOT**	5	25	18.44	5.33

Legend: UA = Upper Airways; LA = Lower Airways; RAI = Respiratory Allergy Impact; GS = Global Score; T5SS = Total 5 Symptoms Score; ACT = Asthma Control Test.

Optimal HRQoL, identified by Rhinasthma Global Summary (GS) score ≤20, was reached by 78/209 (37.32%) (Table 3). Clinical characteristics were compared between patients who had or had not achieved optimal HRQoL; no significant differences were found concerning gender, education, and smoking habits, while younger patients had a better HRQoL (p<0.05). Optimal HRQoL achievement was not associated with rhinitis duration (intermittent vs persistent; χ^2^ = 1.753; p<0.229), rhinitis degree of severity (mild/moderate vs severe; χ^2^ = 0.142; p<0.737) and asthma degree of severity (GINA treatment 1 vs GINA 2, 3, 4, 5; χ^2^ = 3.430; p<0.088).

**Table 3:** RHINASTHMA, T5SS and ACT scores (mean and standard deviations) in patients who achieved or not an optimal HRQoL (Student's t-test; m±sd).

	Patients withGS score ≤20(N = 78)		Patients withGS score >20(N = 131)	
	Mean	Std. Deviation	Mean	Std. Deviation
**RHINASTHMA UA**	26.38	9.72	41.41	9.72
**RHINASTHMA LA**	19.97	7.53	43.21	18.29
**RHINASTHMA RAI**	10.10	7.64	35.90	19.44
**RHINASTHMA GS**	12.36	5.03	40.74	15.31
**T5SS TOT**	4.75	3.27	8.30	3.86
**ACT TOT**	21.98	3.79	16.20	5.07

Legend: UA = Upper Airways; LA = Lower Airways; RAI = Respiratory Allergy Impact; GS = Global Score; T5SS = Total 5 Symptoms Score; ACT = Asthma Control Test.

**Figure 1:**
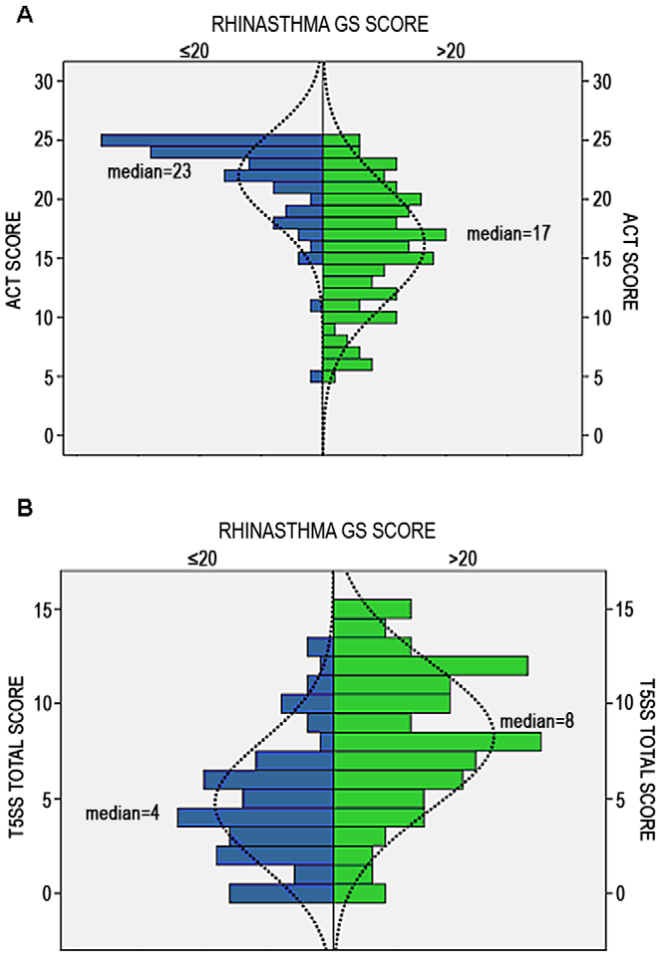
Asthma control and rhinitis symptoms score in patients who had or had not achieved optimal asthma and rhinitis related quality of life (NN-HRQoL). ACT = asthma control test; T5SS = Total 5 symptoms score; GS = global summary. GS score <20 is expression of an optimal asthma and rhinitis related quality of life. In order to describe the ACT (panel A) and T5SS (panel B) variable trend as regards to RHINASTHMA Global Score, a pyramid population graphic was used. Green bars identify patients with GS score >20; blue bars identity patients with GS score <20.

Patients with an optimal GS score showed significantly lower T5SS score (p<0.001) and higher ACT score (p<0.001) compared to those with a GS score higher than 20. ACT score, categorized into uncontrolled (<20) and controlled (≥20) scores, was significantly associated with GS optimal score achievement (χ^2^ = 49.599; p<0.001). Patients achieving optimal HRQoL showed median ACT and T5SS values greater than patients not reaching optimal HRQoL (Figures 1A–B). Considering only controlled patients, the level of ACT score (25 vs 20–24) is associated to the achievement of the optimal HRQoL: a significantly higher percentage of totally controlled patients compared to well controlled ones (84% vs 51%) reached the optimal HRQoL (χ^2^ = 7.617; p<0.006).

Concerning illness perception, patients who did not reach optimal HRQoL reported more symptoms and ascribed more of them to asthma and rhinitis (Table 4), and showed significantly higher identity, illness consequences, timeline acute/chronic - cyclical, and emotional representation scores than patients who achieved optimal HRQoL (Table 5). Linear regression of the IPQ-R scores indicated that identity is the most relevant factor in predicting the RHINASTHMA GS score (Test F = 57.46; p<0.001).

**Table 4:** Illness perception in patients reaching near-normal HRQoL (GS score <20) and not reaching an optimal HRQoL (GS ≥20). (Student's t-test; m±sd).

IPQ-R Symptom	Presence of the symptom			Relation between the symptom and asthma/rhinitis		
	GS ≤20(N = 78)	GS >20(N = 131)	p	GS ≤20N = 78)	GS >20(N = 131)	p
**Pain**	0.17±0.38	0.28±0.45	0.08	0.13±0.33	0.23±0.42	0.07
**Sore Throat**	0.29±0.46	0.47±0.50	0.02*	0.19±0.40	0.38±0.49	0.01*
**Nausea**	0.08±0.28	0.16±0.36	0.15	0.01±0.12	0.10±0.31	0.02*
**Breathlessness**	0.75±0.43	0.85±0.36	0.10	0.73±0.45	0.81±0.39	0.17
**Weight Loss**	0.04±0.20	0.12±0.33	0.06	0.04±0.20	0.10±0.31	0.13
**Fatigue**	0.58±0.50	0.79±0.41	0.00*	0.44±0.50	0.63±0.48	0.01*
**Stiff Joints**	0.08±0.28	0.33±0.47	0.00*	0.04±0.20	0.19±0.39	0.00*
**Sore Eyes**	0.67±0.47	0.71±0.45	0.51	0.64±0.48	0.67±0.47	0.64
**Wheeziness**	0.82±0.38	0.92±0.27	0.03*	0.78±0.41	0.85±0.35	0.21
**Headache**	0.32±0.47	0.54±0.50	0.00*	0.22±0.42	0.40±0.49	0.01*
**Upset Stomach**	0.18±0.39	0.38±0.49	0.00*	0.07±0.26	0.29±0.91	<0.001*
**Sleep Disturbances**	0.33±0.47	0.69±0.46	0.00*	0.26±0.44	0.56±0.50	<0.001*
**Dizziness**	0.14±0.35	0.36±0.48	0.00*	0.10±0.30	0.28±0.45	<0.001*
**Loss Of Strength**	0.47±0.50	0.74±0.44	0.00*	0.33±0.47	0.60±0.49	<0.001*

Legend: IPQ-R = Illness perception Questionnaire Revised version; GS = Global Summary.

* Level of significance p<0.05.

**Table 5:** Illness perception (IPQ-R) and profile of mood state (POMS) in patients reaching an optimal HRQoL (GS score ≤20) and not reaching an optimal HRQoL (GS >20). (Student's t-test; m±sd).

IPQ-R factors	GS ≤20(N = 78)	GS >20(N = 131)	P value
Identity	3.92±2.46	5.87±3.09	<0.001*
Timeline Acute/Chronic	18.61±5.80	19.94±4.75	<0.001*
Consequences	12.74±4.84	16.17±4.85	<0.001*
Personal Control	19.07±5.31	19.07±4.21	1.00
Treatment Control	17.13±4.18	17.00±2.96	0.80
Illness Coherence	15.68±4.22	14.69±3.92	0.10
Timeline Cyclical	10.84±4.12	12.79±3.63	<0.001*
Emotional Representation	11.78±5.58	15.56±6.11	<0.001*
**POMS factors**			
Tension-Anxiety	45.85±8.12	54.94±12.67	0.001*
Depression-Dejection	47.29±7.02	53.83±12.14	0.001*
Anger-Hostility	47.61±7.55	55.19±13.64	0.001*
Vigor-Activity	51.90±9.99	50.30±11.08	0.350
Fatigue-Inertia	46.97±7.33	56.27±12.31	0.001*
Confusion-Bewilderment	43.58±8.09	51.81±11.30	0.001*
POMS TOT	179.40±37.74	221.74±60.85	0.001*

Legend: IPQ-R = Illness perception Questionnaire Revised version; GS = Global Summary.

* Level of significance p<0.05.

Regarding profiles of mood state, patients who achieved optimal HRQoL reported significantly lower scores in all POMS factors except for Vigor-Activity (Table 5). ROC analysis indicated that the probability for patients to achieve optimal HRQoL was 67% with a cut-off score for POMS of 187.5 (Area under the Curve - AUC = 0.715, sensitivity 69%).

A sub-analysis including the 86 patients with well and totally controlled asthma (ACT score between 20 and 25) was performed. Controlled patients showed significant differences in illness perception and profile of mood states based on whether patients had achieved optimal HRQoL (Table 6).

**Table 6:** Patient reported outcomes (PROs) differences in patients with controlled asthma (ACT ≥20) (N = 86) achieving or not an optimal HRQoL. (Student's t-test; m±sd).

IPQ-R factors	GS ≤20(N = 51)	GS >20(N = 35)	P value
Identity	3.80±2.458	5.57±2.515	0.003*
Timeline Acute/Chronic	19.62±4.802	20.10±3.933	0.645
Consequences	12.42±4.348	14.97±3.567	0.008*
Personal Control	19.82±4.715	19.40±3.013	0.664
Treatment Control	17.60±3.580	17.23±2.800	0.633
Illness Coherence	16.56±3.308	16.10±2.905	0.531
Timeline Cyclical	10.98±3.473	11.63±3.662	0.427
Emotional Representation	11.66±4.684	13.45±5.650	0.126
**POMS factors**			
Tension-Anxiety	45.09±8.479	54.63±11.943	0.001*
Depression-Dejection	46.88±6.470	52.85±11.292	0.006*
Anger-Hostility	46.86±6.151	54.41±13.582	0.002*
Vigor-Activity	52.74±9.587	54.15±10.582	0.569
Fatigue-Inertia	47.21±7.117	55.11±11.318	0.001*
Confusion-Bewilderment	43.05±7.329	50.67±11.156	0.001*
POMS TOT	176.35±34.474	213.52±61.296	0.002*

Legend: IPQ-R = Illness perception Questionnaire Revised; POMS = Profile of Mood States Questionnaire. GS = global summary. A GS score <20 is expression of an optimal asthma and rhinitis related quality of life.

* Level of significance p<0.05.

## Discussion

The goals of this observational real-life study were to verify whether the clinical-trial HRQoL results were achievable in a real-life setting also considering the burden of comorbid rhinitis, and to explore which disease- and patient-related factors were associated with this achievement. Our study shows that optimal HRQoL is an achievable target but is rarely achieved in real life in patients suffering from allergic asthma with comorbid rhinitis.

With the exception of age, the patient demographic characteristics were not associated with the achievement of optimal HRQoL; indeed, our results are in line with previous findings indicating a reduced HRQoL in older subjects [20], [21]. Possible explanations for this finding include the longer duration of symptoms in older persons and the way they face their disease Optimal HRQoL achievement was independent of the degree of severity of asthma and rhinitis, an observation in agreement with previous clinical results [3], [6], [22]. In line with previous studies [3], [11] our results confirm that, in asthma rhinitis patients observed in real life, totally controlled patients were more likely to reach optimal HRQoL, and patients were able to distinguish between well and total control in terms of HRQoL [3].

Patients reaching an optimal HRQoL differed in disease perception and mood (Tables 4, 5 and 6). They enjoy a clearer vision of the symptomatological characteristics of the illness and awareness of its temporal evolution, attributing fewer negative life consequences and fewer unpleasant emotions to the disease. The association between illness perception and HRQoL can lead to two different outcomes. On one hand, reaching a optimal HRQoL can result in a better vision of the disease itself, with the patient perceiving the disease as more integrated in daily life and less burdensome. On the other hand, disease perception itself contributes to its impact on life. In fact, the number of symptoms that patient ascribes to respiratory allergy (identity factor) is the strongest IPQ-R correlate of HRQoL (RHINASTHMA GS score).

Therefore, investigating which symptoms and disturbances patient associates with their disease, providing a correct education, helping them to recognize the symptomatological characteristics of asthma and rhinitis and to distinguish them from symptoms related to other pathologies, seems a necessary step in order to reach and maintain optimal HRQoL levels. Patients attaining optimal HRQoL reported lower levels of tension, depression, aggressiveness, tiredness, and confusion (Table 5). If the patients' HRQoL was not bothersome, the presence of the disease would not influence their mood, although patients with a better mood profile could possess more psychological resources to cope with their disease. The outcomes measures that describe patients' subjective viewpoint are increasingly emphasized as an essential indicator in asthma [12], [23].

Asthma treatment has been shown to greatly highly improve HRQoL and reduce the burden of asthma [1], [2]; moreover, total control of asthma is an achievable goal that patients perceive in terms of quality of life. However, real-life surveys indicated that rhinitis symptoms, detectable in most asthmatics, were often underestimated, and asthma control was often not reached [10], [11]. In our study, the level of control was significantly associated with HRQL; moreover, well-controlled and totally controlled patients significantly differed in achieving optimal HRQoL. Among controlled patients, those who reach an optimal HRQoL ascribe less symptoms to the disease, reporting less consequences of asthma and rhinitis in their life and showing a better mood profile. Our results should be evaluated in the light of study's strengths and limits. The main strength is that data collection was conducted in real life, providing relevant data for clinical management of asthma and comorbid rhinitis. Several limitations should be taken into account. First of all the small number of patients enrolled in the study and second, cross sectional nature of the analysis does not allow for drawing causal inferences of the determinants of optimal HRQoL. Third, due to the study design, we were unable to control for the confounding effects such as prescribed drugs, treatment adherence, and presence of physical or psychiatric diseases.

Although clinical trials have demonstrated that asthmatic patients may reach an optimal HRQoL, which implies minimal or absent disease impact on patient life, this possibility had not been explored yet in a real-life setting. The present study demonstrates that, although the majority of real-life patients with both asthma and rhinitis did not achieve optimal HRQoL, this is an achievable target. Failure to reach optimal HRQoL is related neither to disease severity nor to demographic characteristics (apart from age). Optimal HRQoL is related to asthma control and rhinitis symptoms and a significantly higher percentage of totally controlled patients reached an optimal HRQoL level. This evidence, confirming the results of clinical trials [4], [6], could be useful in targeting treatment goals in clinical practice. Impaired HRQoL may be an unnecessary hardship in some patients that may be avoided by aiming for best-achievable control of both asthma and rhinitis. This objective may be reached through a treatment plan tailored according to both disease and patient-related factors. Further research using longitudinal design with larger sample sizes are required to better explore the causal relationship between optimal HRQoL achievement and patient and disease related factors.
